# Patellar Avulsion Fracture

**DOI:** 10.7759/cureus.46695

**Published:** 2023-10-08

**Authors:** Rohan Potla, Tovah Williamson, Sidhartha R Ramlatchan, Rohan K Mangal, Latha Ganti

**Affiliations:** 1 Biomedical Sciences, University of Central Florida, Orlando, USA; 2 Medicine, University of Central Florida College of Medicine, Orlando, USA; 3 Chemistry, Drexel University, Philadelphia, USA; 4 Medicine, University of Miami Miller School of Medicine, Miami, USA; 5 Medical Sciences, The Warren Alpert Medical School of Brown University, Providence, USA; 6 Emergency Medicine & Neurology, University of Central Florida College of Medicine, Orlando, USA

**Keywords:** kirschner wire, open reduction and internal fixation (orif), patella fracture, avulsion fracture, patella

## Abstract

We report on the case of a 52-year-old male who sustained a transverse patellar fracture after tripping on uneven pavement. These fractures can be easy to miss on anteroposterior views, highlighting the importance of multiple radiographic views of the knee. Examination of the knee is also important, as initial clinical appearance can be benign. These fractures are most often seen in adolescents, which makes the current case somewhat unusual.

## Introduction

The patella is a critical bone in the articulation between the quadriceps and patellar tendons and serves as the lever for the extensor mechanism of the knee [1]. Overall, patellar fractures are rare, comprising only 1% of all skeletal fractures [2]. Patellar avulsion fractures are injuries in which a fragment of the patella detaches from the main bone due to the excessive pull of the quadriceps tendon or patellar ligament. They are relatively rare compared to other types of patellar fractures. They often occur in adolescents and young adults, with a higher prevalence in males [3]. The reason for the predilection for younger age is that bones are still developing in adolescents, and also because they are more involved in contact sports that lend themselves to forceful contractions of the quadriceps such as in jumping or kicking.

On radiographs, lateral views are useful to assess the displacement of the patellar fragments [4]. The management of patellar avulsion fractures depends on the size of the fracture fragment, the degree of displacement, and the patient's age [5,6]. Conservative management is generally reserved for small, non-displaced fractures, and consists of immobilization, protected weight-bearing, and physical therapy. Larger, displaced fractures or fractures in younger patients often require open reduction and internal fixation (ORIF) to reattach the fragment using screws, wires, or sutures, which are thought to reduce surgical time [7]. Physical therapy is crucial in both conservative and surgical management to regain strength, mobility, and function of the knee. Complications of patellar avulsion fractures are those common to many orthopedic fractures, including nonunion or malunion, knee stiffness [8], hardware that is painful [9], migration of wires [10], patellar instability, and chondral damage. In some cases, patellar instability can lead to recurrent patellar dislocation or subluxation, and damage to the cartilage can lead to osteoarthritis over time.

## Case presentation

**Figure 1:**
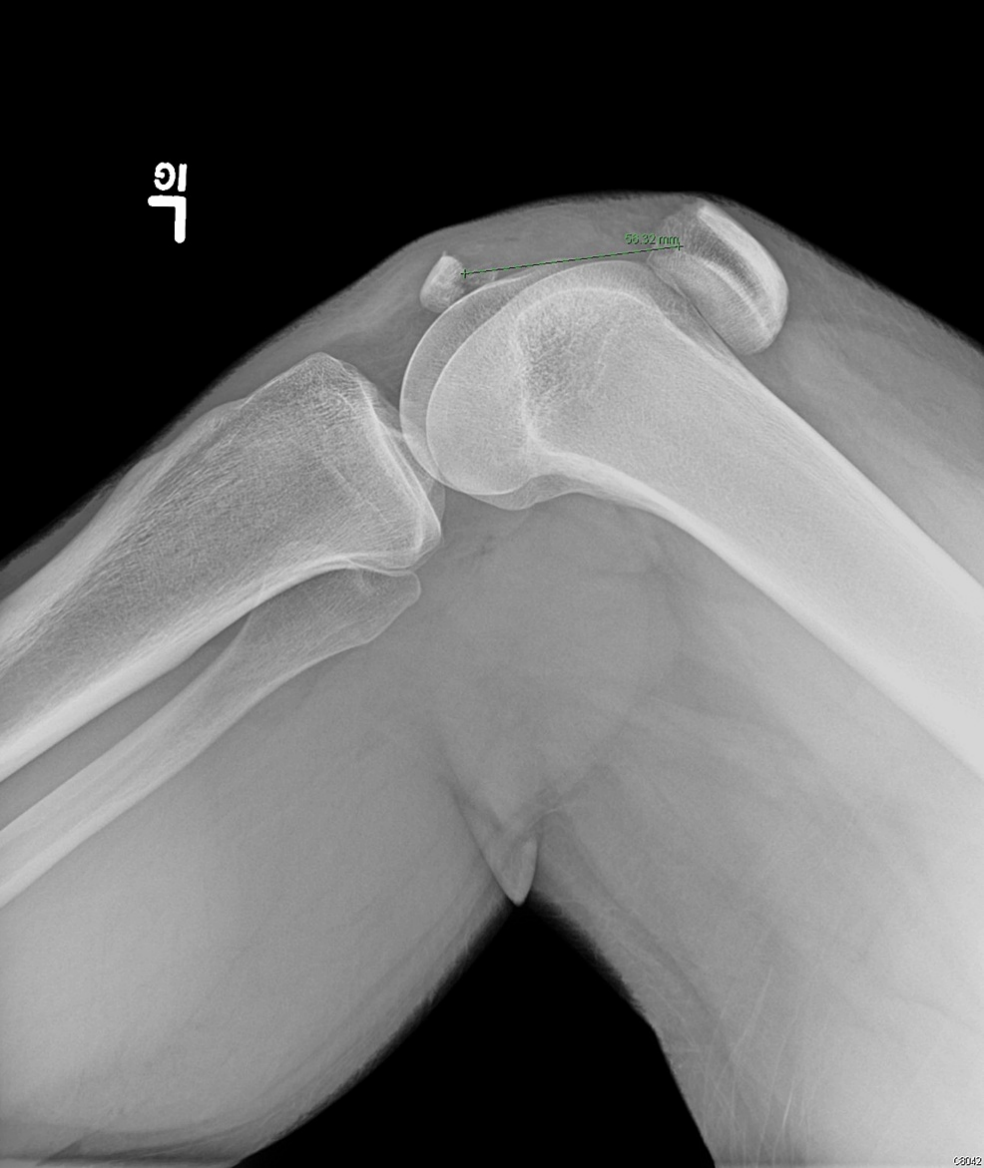
Radiograph of the lateral view of the left knee demonstrating patellar fracture with 56 mm of distraction of fracture fragments

A 52-year-old male presented to the Emergency Department due to knee pain. He had tripped on uneven concrete, landing on his left knee. His vital signs were a temperature of 98.7°F, pulse of 92 beats per minute, blood pressure of 161/92 mmHg, respiration of 18 breaths per minute, and oxygen saturation of 97% on room air. The patient did not have any other complaints. He did not injure any other part of his body. Physical examination of the knee revealed impaired ability to fully extend the knee or perform a straight leg raise. While there was no open wound, ecchymosis over the anterior knee at the site of impact was present. The patient did not have a sensory deficit. Palpation of the knee revealed a divot that corresponded to the gap between the fracture fragments. Radiographs of the knee revealed a transverse patellar avulsion fracture on lateral view with over 56 mm between the fracture fragments (Figure 1).

**Figure 2:**
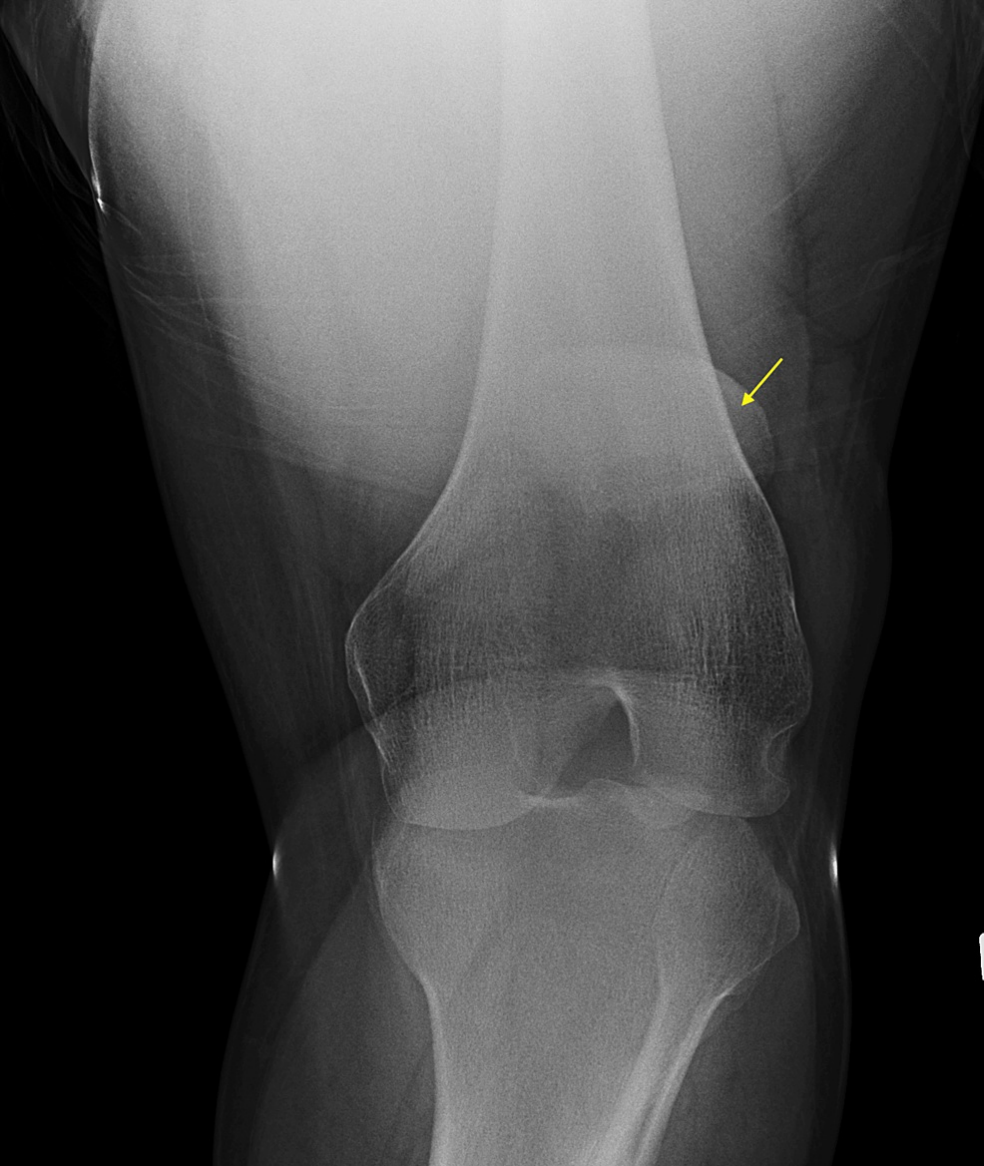
Radiograph depicting an anteroposterior view of the patella (arrow)

Notably, the fracture was barely noticeable on the anteroposterior view (Figure 2).

The patient was given intravenous ketorolac for analgesia. Orthopedics was consulted. The fracture was repaired using a tension band in a "figure-of-eight" configuration. The patient followed up with physical therapy as an outpatient and was doing well at a two-week follow-up.

## Discussion

Avulsion fractures of the patella in adults more commonly originate at the patellar tendon, though the most common origin overall is at the inferior pole of the patella, as seen in the pediatric population [11]. The patient in question, a white male in his early 50s, was reported to work in construction and fell on uneven pavement. There are multiple classification types for patellar fracture, which include but are not limited to descriptive classifications based on fracture pattern. The Ortiguera and Berry classification evaluates stability and suggests operative management depending on the type and the AO/OTA (Arbeitsgemeinschaft fur Osteosynthesfragen) classification is based on the articular involvement [4].

This case can be classified as a 34-A (extra-articular) avulsion fracture, minimally a type IIIa Ortiguera and Berry classification (loose patellar component with reasonable bone stock), which typically requires surgical management [4]. Patellar avulsion fractures normally occur in pediatric populations due to an increased ratio of the strength of tendon and muscle to osseocartilaginous structures; however, the mechanism of injury and patient circumstances potentially explain the uncommon patient demographics in this case. 

During the primary evaluation, a good history of injury mechanism, as well as a physical exam of the knee stability and assessment of extension/flexion are important steps. Imaging of patellar fractures is typically plain radiographs primarily. Lateral knee radiographs are more useful than anteroposterior view radiographs in evaluating the pathology of the knee due to the difficulty of discerning structures that are superimposed [12]. A 2022 study showed similar outcomes and a high number needed to treat (NNT) with the addition of oblique views, and avoiding such views was discussed to reduce radiation load and hospital costs [13]. Additional CT imaging is useful in optimizing management, especially prior to surgical interventions [2]. MRI can be useful in understanding articular and tendinous involvement in patellar fractures [5].

For displaced fractures greater than 2-3 mm of step-off and greater than 1-4 mm fracture gap, surgical management is required to restore extensor function. ORIF was indicated in this patient due to loss of extensor function [5]. Patellar fractures of this nature can be managed with a variety of surgical techniques and material components, the most common being the modified anterior tension band technique which uses an 18-gauge stainless-steel wire in a figure-of-eight configuration around two K-wires (Kirschner wires). Additional cerclage around the patella is recommended for comminuted fractures [14]. Other materials can be used, such as ultra-high-molecular-weight polyethylene (FiberWire), which has been shown to have better outcomes in certain types of fractures [15,16]. A recent randomized controlled trial discussed the use of headless screws in cadaver knees and showed comparable outcomes to cannulated screws for fixation strength and subfailure fragment displacement. Headless screws can improve long-term outcomes in patients as the screw head can lead to adjacent structure erosion; however, additional studies are needed to confirm efficacy.

## Conclusions

Patellar avulsion fractures in the adult population are uncommon, but knowledge of proper evaluation and management is nevertheless valuable. This 52-year-old male presented with a closed transverse patellar avulsion fracture with no articular involvement, seen best on lateral plain radiograph. He required ORIF, which was done with the traditional AO method with a stainless-steel wire and two K-wires. Other patellar fracture types can be managed conservatively, and awareness of classifications, methodology, and differences in treatment are paramount.
